# Research of Method for Solving Relaxation Modulus Based on Three-Point Bending Creep Test

**DOI:** 10.3390/ma12122021

**Published:** 2019-06-24

**Authors:** Yazhen Sun, Zhangyi Gu, Jinchang Wang, Xuezhong Yuan

**Affiliations:** 1 School of Transportation Engineering, Shenyang Jianzhu University, Shenyang 110168, China; 2 College of Civil Engineering and Architecture, Zhejiang University, Hangzhou 310058, China; guzhangyi315@163.com; 3 Institute of Transportation Engineering, Zhejiang University, Hangzhou 310058, China; wjc501@zju.edu.cn; 4 School of Science, Shenyang Jianzhu University, Shenyang 110168, China

**Keywords:** HVAS, transcendental equation, relaxation modulus, creep compliance, convolution, Newton’s method, Boltzmann superposition principle

## Abstract

A method was developed for solving the relaxation modulus of high viscosity asphalt sand (HVAS) based on the three-point bending creep test, and was verified by comparison with experimental results. In this method, firstly, a transcendental equation was obtained by the convolution, and then equations were obtained by Taylor’s formula, which were solved by Mathmatica to obtain the relaxation modulus by Newton’s method. Subsequently, the laboratory investigations of the viscoelastic parameters of the Burgers model for the HVAS by three-point bending creep tests were carried out. In addition, the method was verified by comparing the relaxation moduli with the indoor relaxation experiments. Results showed that the numerical calculation and the test data were in good agreement, and the relaxation characteristics of the HVAS were reflected more accurately. The method can be used to study the relaxation characteristics of the asphalt mixtures effectively. In addition, this study provides a research basis for road crack prevention.

## 1. Introduction

The pavement performance and durability of the asphalt mixture road will be seriously affected after pavement cracking [1,2,3]. The reason is that the road spans a vast area, where the range of temperature could be very large due to environmental changes, so the inconsistency of deformation between the surface and the base causes thermal stress [4,5,6]. If the relaxation ability is strong, the thermal stress is released so that it does not exceed the allowable value. Relaxation modulus is an important parameter for analyzing and evaluating relaxation ability [7]. Therefore, simple and accurate calculation of relaxation modulus is an important basis for the study of viscoelastic parameters of the asphalt mixtures, which provides the research basis for road crack prevention.

The relaxation modulus could be directly obtained from relaxation test [8,9,10]. However, constant strain should be applied to the specimen instantaneously and remains unchanged during the relaxation test, which poses a great challenge to instrument selection, test design and operation [11]. Higher hardness of asphalt mixture at low temperature leads to longer relaxation time, which also increases the difficulty of the relaxation test [12]. Therefore, direct measurement of the relaxation modulus by the relaxation test often results in large errors, and it is difficult to operate. By contrast, a creep test is easier to be carried out, so solving *E*(*t*) by *J*(*t*) was one of the focuses of the researches [13,14,15]. In such case, apart from the relaxation tests, the interconversion of the creep compliance was recommended [16].

It is to be noted that in a rheological application, the convolution of *J*(*t*) and *E*(*t*) for the LVEs is expressed according to the Boltzmann superposition integral [17] as

(1) $$\int_{0}^{t}J\left(t-\tau \right)E\left(\tau \right)d\tau =t$$

where *J*(*t*) is the creep compliance function, *E*(*t*) is the relaxation modulus function and *t* is the time.

In the early application of the integral, the relation of the approximate *J*(*t*) and *E*(*t*) were usually taken as reciprocal, being only applicable to weakly viscous materials instead of viscoelastic materials. Lately, with the introducing of several variables [18], it is possible to derive an inverse reciprocal relation, and only when *t* = 0 or *t* = ∞, the reciprocal relation applies [19], i.e.,

(2) $$\left\{ \begin{array}{l} t=0\;E\left(0\right)=1/J(0)\\ t=\infty \;E\left(\infty \right)=1/J(\infty ) \end{array}\right.$$

With the increasing need of interconversion between the LVE functions of the HVAS, approaches were proposed to solve the interconversion equation between *J*(*t*) and *E*(*t*). For example, a numerical integration method for determining of *E*(*t*) was proposed according to the measured *J*(*t*) based on the accurate approximate transformation between the volume *J*(*t*) and the bulk relaxation modulus [20]; a numerical interconversion based on the convolution integral, which relates *E*(*t*) to *J*(*t*), was made to discrete the time domain into a finite number of sub domains, and an iterative expression was obtained [21]. However, in the abovementioned methods, *E*(*t*) depended on the accuracy of the numerical calculation; this led to large differences in the calculations with the test data when the computational accuracy was poor. As noted by some authors [13,22,23], *J*(*t*) and *E*(*t*) were substituted into integral relationship simplified as a matrix form, and the Dirac Delta function was introduced to solve the creep compliance. With respect to the integrals expressed in the Prony series, a poorly conditioned matrix equation increases the complexity of the calculation.

Many scholars have studied and some achievements have been made in determining the relaxation modulus of the asphalt mixture by using the complex modulus. Zhao determined the main curve of the storage moduli by using the test results of complex moduli of the asphalt mixture, and converted the storage moduli into the relaxation moduli of asphalt mixture by using the collocation method and related viscoelastic theoretical formula [24]. On the basis of the analysis of previous findings, Liu gave the relationship between the complex moduli and the relaxation moduli [14]. However, in determining the complex moduli of asphalt mixtures, tests need to be carried out at several temperatures. Moreover, at each temperature, the complex moduli of the asphalt mixtures with different angular frequencies need to be measured to obtain the dynamic moduli and phase angle of asphalt mixtures.

In this paper, a new scheme is adopted to solve the convolution formula. Previous studies have used the same model to characterize the relaxation and creep of asphalt at the same time [19,25,26]. Common models, such as the Burgers model, Maxwell model and Kelvin model, are good at creep and relaxation alone, but it is very difficult to take both into account [27,28]. Therefore, the advantages of the Burgers model and Generalized Maxwell model (GMM) are integrated in their respective fields to better characterize the properties of the HVAS. A more general formula not confined to the form of the Prony series is introduced and the solution process is also simplified. First, *J*(*t*) was obtained based on the three-point bending creep tests. Then, by substituting the parameters for the Burgers model into the convolution integral expanded by the Taylor series, *E*(*t*) was derived from Wolfram Mathematica 8 using Newton’s method. For verifying, the relaxation tests were carried out and the *E*(*t*) obtained was compared with that determined by the suggested approach. Moreover, with the application of the time-temperature equivalence principle, the master curve of *E*(*t*) was obtained within a larger temperature range.

This method is not only straightforward and accurate in the interconversion (without the application of Laplace transform) between the compliance and the modulus functions, but can also avoid the high requirements for experiment equipment. Therefore, the method is recommended to study the relaxation characteristics of the HVAS.

## 2. Interconversion of Creep Compliance and Relaxation Modulus

### 2.1. Interconversion Equation

**Figure 1:**
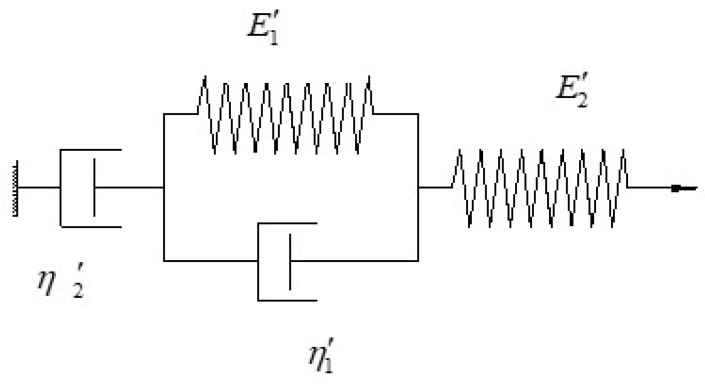
Burgers model.

When a constant stress is applied to the asphalt mixture, the development of the corresponding strain can be divided into three stages, i.e., the instantaneous elastic stage, the viscous flow stage and the delayed elastic deformation stage. The creep behaviour of the HVAS can be characterized with the four-parameters ($E_{1}^{\prime }$, $E_{2}^{\prime }$, $\eta_{1}^{\prime }$, $\eta_{2}^{\prime }$) of the Burgers model, as shown in Figure 1.

The creep compliance *J*(*t*) for the Burgers model of the HVAS is written as:

(3) $$J(t)=\frac{1}{E_{1}^{\prime }}+\frac{t}{\eta_{1}^{\prime }}+\frac{1}{E_{2}^{\prime }}(1-e^{-\frac{E_{2}^{\prime }}{\eta_{2}^{\prime }}t})$$

where $\eta_{1}^{\prime }$ and $\eta_{2}^{\prime }$ are the viscosity coefficients in the Burgers model, $E_{1}^{\prime }$ and $E_{2}^{\prime }$ are the elastic moduli in the Burgers model.

**Figure 2:**
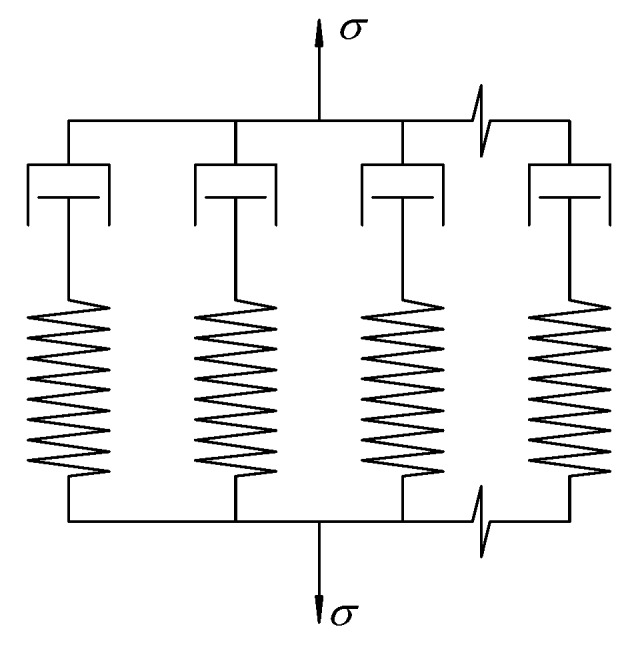
Generalized Maxwell model (GMM).

A reliable performance prediction model is conducive to the development of the relaxation behaviour [29]. To this end, the Generalized Maxwell model (GMM) assembled by parallel Maxwell elements representing the relaxation module is employed, as shown in Figure 2. The overall relaxation modulus is

(4) $$E\left(t\right)=\overset{n}{\underset{i=1}{\sum }}E_{i}e^{-\frac{t}{\tau_{i}}}$$

where *i* is the *i*-th spring-dashpot element, *i* is the relaxation time (the higher its value, the longer it takes for the strain/stress to relax) related to the dashpot viscosity, *E_i_* is the modulus of the *i*-th Maxwell element associated to the spring stiffness, *n* is the number of spring-dashpot elements accounted for and *t* is the time.

Substituting Equation (3) and (4) into the convolution integral of Equation (1), we obtain

(5) $$\int_{0}^{t}\left[\sum_{i=1}^{n}E_{i}e^{-\frac{\tau }{\tau_{i}}}\right]\left[\frac{1}{E_{1}^{\prime }}+\frac{t-\tau }{\eta_{1}^{\prime }}+\frac{1}{E_{2}^{\prime }}(1-e^{-\frac{E_{2}^{\prime }}{\eta_{2}^{\prime }}(t-\tau )})\right]d\tau =t$$

### 2.2. Derivation of Relaxation Modulus

The target number of spring-dashpot elements *n* is predefined before solving. After the expansion of the integral, Equation (5) can be re-rewritten in terms of *E_i_*, *τ_i_*, etc., as:

(6) $$\begin{array}{l} \left(\frac{1}{E_{1}^{\prime }}+\frac{1}{E_{2}^{\prime }}\right)\sum_{i=1}^{n}E_{i}\tau_{i}-\left(\frac{1}{E_{1}^{\prime }}+\frac{1}{E_{2}^{\prime }}\right)\sum_{i=1}^{n}E_{i}\tau_{i}e^{-\frac{t}{\tau_{i}}}+\sum_{i=1}^{n}\frac{E_{i}}{\eta_{1}^{\prime }}\tau_{i}t-\sum_{i=1}^{n}\frac{E_{i}}{\eta_{1}^{\prime }}\tau_{i}+\\ \sum_{i=1}^{n}\frac{E_{i}}{\eta_{1}^{\prime }}e^{-\frac{t}{\tau_{i}}}\tau_{i}^{2}-\frac{1}{E_{2}^{\prime }}\sum_{i=1}^{n}E_{i}\frac{\tau_{i}\eta_{2}^{\prime }}{\eta_{2}^{\prime }-\tau_{i}E_{2}^{\prime }}e^{-\frac{E_{2}^{\prime }}{\eta_{2}^{\prime }}t}+\frac{1}{E_{2}^{\prime }}\sum_{i=1}^{n}E_{i}e^{-\frac{t}{\tau_{i}}}\cdot \frac{\tau_{i}\cdot \eta_{2}^{\prime }}{\eta_{2}^{\prime }-\tau_{2}E_{2}^{\prime }}=t \end{array}$$

where *E_i_* (*i* = 1 to 6) is the elastic modulus in GMM, *τ_i_* (*i* = 1 to 6) is the relaxation time in GMM, $\eta_{1}^{\prime }$ and $\eta_{2}^{\prime }$ are the viscosity coefficients in the Burgers model, $E_{1}^{\prime }$ and $E_{2}^{\prime }$ are the elastic moduli in the Burgers model. Actually, this is a transcendental equation. Although the commands Solve and NSolve in Mathematica can be applied to solve exponential and trigonometric equations in a limited way, they are not designed to solve complicated transcendental equations. Fortunately, Equation (6) can be solved by Mathematica using Taylor’s formula.

#### 2.2.1. Taylor’s Formula

To simplify the calculations, multinomials obtained by Taylor’s formula were used in the alternative method. The application of the multinomials in the study of the HVAS is a progress, especially in the computer program field, which can be done with high precision and is an applicable approximate method for the calculations of the complex functions. Taylor’s formula with a surplus item Peano meets *n*-order differentiable at the point *x*_0_ [30]. In other words, this type of function, with a simple form and widely applicable conditions, is very convenient to deal with some qualitative problems. Due to Equation (6), *n*-order derivative exists at 0, Taylor’s formula with a surplus item Peano is used to expand at 0, namely, the Maclaurin formula. In our study, there are 12 unknown parameters of the generalized Maxwell model, so the equation is expanded to 12 polynomials about *t* and a Peano remainder, and the required accuracy of the calculations can be acquired. We have:

(7) $$\begin{array}{l} \left(E_{1}\tau_{1}+E_{2}\tau_{2}+E_{3}\tau_{3}+E_{4}\tau_{4}+E_{5}\tau_{5}+E_{6}\tau_{6})(\frac{1}{E_{1}^{\prime }}+\frac{1}{E_{2}^{\prime }}-\frac{1}{\eta_{1}^{\prime }}+1\right)\\ -\frac{\eta_{2}^{\prime }}{E_{2}^{\prime }}\left(\frac{E_{1}\tau_{1}}{\eta_{2}^{\prime }-E_{2}^{\prime }\tau_{1}}+\frac{E_{2}\tau_{2}}{\eta_{2}^{\prime }-E_{2}^{\prime }\tau_{2}}+\frac{E_{3}\tau_{3}}{\eta_{2}^{\prime }-E_{2}^{\prime }\tau_{3}}+\frac{E_{4}\tau_{4}}{\eta_{2}^{\prime }-E_{2}^{\prime }\tau_{4}}+\frac{E_{5}\tau_{5}}{\eta_{2}^{\prime }-E_{2}^{\prime }\tau_{5}}+\frac{E_{6}\tau_{6}}{\eta_{2}^{\prime }-E_{2}^{\prime }\tau_{6}}\right)\\ +(-\frac{E_{1}\tau_{1}+E_{2}\tau_{2}+E_{3}\tau_{3}+E_{4}\tau_{4}+E_{5}\tau_{5}+E_{6}\tau_{6}}{\eta_{1}^{\prime }}+\frac{E_{1}\tau_{1}}{\eta_{2}^{\prime }-E_{2}^{\prime }\tau_{1}}+\frac{E_{2}\tau_{2}}{\eta_{2}^{\prime }-E_{2}^{\prime }\tau_{2}}+\frac{E_{3}\tau_{3}}{\eta_{2}^{\prime }-E_{2}^{\prime }\tau_{3}}+\frac{E_{4}\tau_{4}}{\eta_{2}^{\prime }-E_{2}^{\prime }\tau_{4}}\\ +\frac{E_{5}\tau_{5}}{\eta_{2}^{\prime }-E_{2}^{\prime }\tau_{5}}+\frac{E_{6}\tau_{6}}{\eta_{2}^{\prime }-E_{2}^{\prime }\tau_{6}}+E_{1}+E_{2}+E_{3}+E_{4}+E_{5}+E_{6}+1)t+\cdot \cdot \cdot +O{\left[t\right]}^{12}\;=\;0 \end{array}$$

where *E_i_* (*i* = 1 to 6) is the elastic modulus in GMM, *τ_i_* (*i* = 1 to 6) is the relaxation time in GMM, $\eta_{1}^{\prime }$ and $\eta_{2}^{\prime }$ are the viscosity coefficients in the Burgers model, $E_{1}^{\prime }$ and $E_{2}^{\prime }$ are the elastic moduli in the Burgers model and the ellipsis “∙∙∙” represents a total of 11 items.

#### 2.2.2. Solutions of Transcendental Equation

For this kind of problem, the command FindRoot in Mathematica can be used. The kernel algorithm of FindRoot is the iterative Newton’s method [31]. In the calculation, the first or the first two points (the initial guess) should be specified, for the best results, the initial guess should be as close to the expected root as possible.

By default, 15 iterations are performed before FindRoot is aborted. The number of iterations is controlled by the Max Iterations, which can be used to increase the number of iterations to obtain more accurate values and to prevent early termination of operations before the desired results are obtained.

For example, to improve the accuracy, the FindRoot command in Mathematica can be written as [*lhs* = = *rhs*, {*x*, *x*_0_, *x*_min_, *x*_max_}, Max Iterations → 200]. Here, the equation solved is *lhs* = = *rhs*, 200 iterations will be carried out in the interval [*x*_min_, *x*_max_], and the roots will be found near *x*_0_. In order to solve the unknowns of *E_i_* and *τ_i_* in Equation (7), the bending creep tests were carried out.

## 3. Creep Tests and Calculations of Relaxation Moduli

### 3.1. Three-Point Bending Creep Tests

#### 3.1.1. Material Properties

The bitumen with 70 penetrations is used as the asphalt binder for the samples. Limestones are used as the aggregates. The optimum ratio of oil to aggregate is 8.1%. The continuous aggregate gradation has a nominal maximum size of 10 mm. An additional 0.7% TCA (temperature controlling viscosity acid) additive of the asphalt mixture mass and 1% activated rubber crumb and 0.7% TCA additive of the mass of the asphalt mixture were added during the blending process.

#### 3.1.2. Sample Preparations

**Figure 3:**
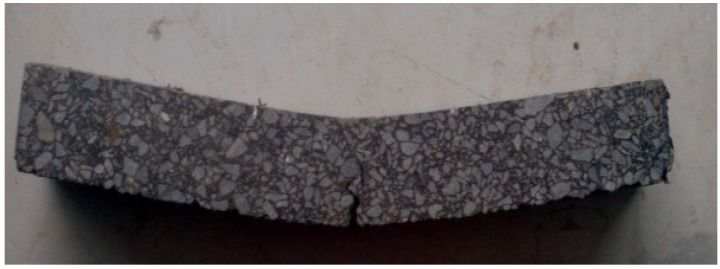
Specimen for bending creep test.

**Figure 4:**
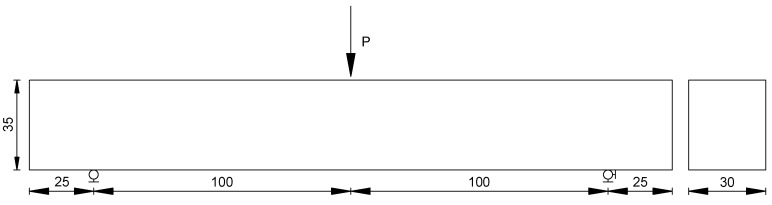
Dimensions of three-point bending beam (mm).

The track plate samples made by wheel rolling which agree with the standard test method [32], were cut into beams with dimensions of 250 × 30 × 35 mm^3^ (Figure 3), the effective span of the beam is 200 mm, as shown in Figure 3 and Figure 4.

#### 3.1.3. Bending Creep Tests Procedure

**Figure 5:**
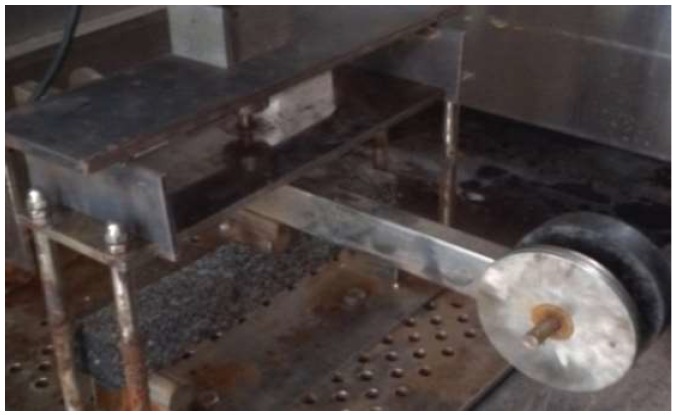
Bending creep test.

The creep test is a test method for determining the viscoelastic parameters of materials, which is commonly used to evaluate the creep properties of materials. The effects of temperatures on the creep compliance were investigated. The bending creep tests were carried out at three temperatures of 0 °C, −5 °C and −15 °C, respectively. After the specimens were put into the environmental chamber, the temperature was increased to the expected value. In order to reach thermal equilibrium in the specimens, it was conditioned for over 4 h, after that, a stress of 10% of the failure load was applied according to the standards. The creep time was designed as 4 h. The lever-loading device applied the load and the displacement meter was used to measure the center displacement of the beam, as shown in Figure 5. The experiment was repeated three times, and the averages were determined by Equations (8)–(10) given below.

The bending tension stress *σ*_0_ is calculated as:

(8) $$\sigma_{0}=\frac{3LP_{0}}{2bh^{2}}$$

where *P*_0_ is the concentrated force applied at the middle of the specimen (in N), *b* is the width of the specimen section (in m), *h* is the height of the cross section of the specimen (in m) and *L* is the span of the specimen (in m).

The bending tension strain is calculated as:

(9) $$\varepsilon (t)=\frac{6hd(t)}{L^{2}}$$

where *d*(*t*) is the midspan deflection which varies with the time *t* during the loading (in m).

The bending creep compliance is calculated as:

(10) $$J(t)=\frac{\varepsilon (t)}{\sigma_{0}}$$

#### 3.1.4. Test Results and Calculation of Creep Compliance

**Figure 6:**
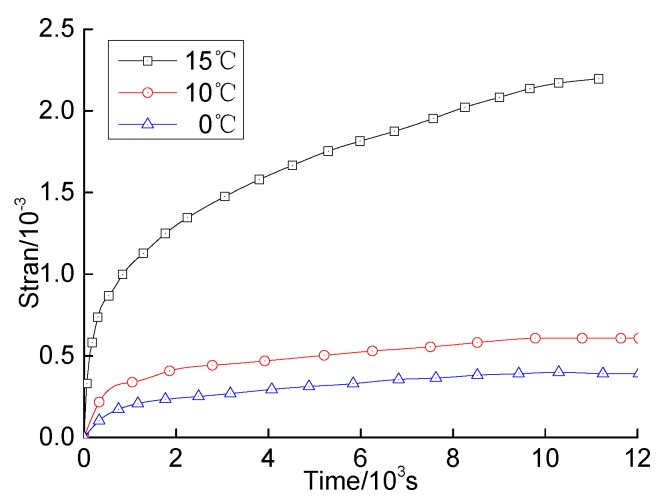
Bending tension strain compared to time at temperatures 0 °C, 10 °C and 15 °C.

The bending strains of the specimens changing with the time at different temperatures under the same stress level are shown in Figure 6.

**Figure 7:**
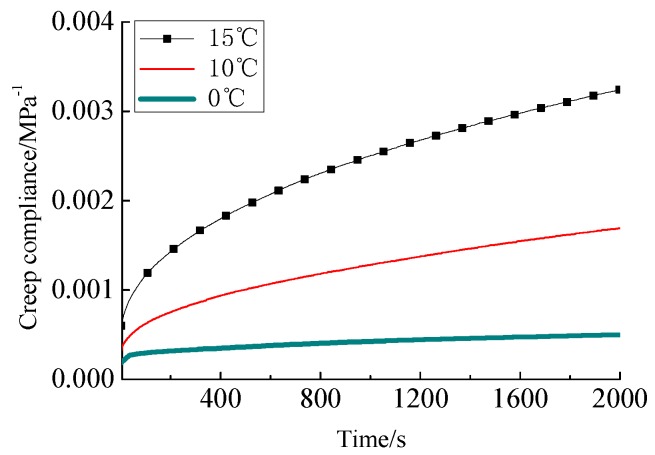
Creep compliances at temperatures 0 °C, 10 °C and 15 °C.

As shown in Figure 6 and Figure 7, the creep process of the HVAS can be divided into three stages, i.e., the initial creep stage, the stable creep stage and the accelerating creep stage. The creep rates increase with the temperature.

According to Equation (10), the stress and strain can be used to determine the creep compliance. The creep compliances at different temperatures are shown in Figure 7.

### 3.2. Determination of Model Parameters

Mechanical models may be considered as different combinations of linear spring(s) and linear dashpot(s) in various series and/or parallel arrangements depending upon the complexity of viscoelastic material behaviour. These basic elements and their combinations allow the better modelling of the viscoelastic behaviour of the asphalt mixtures and the binders than the empirical mathematical models. The linear spring response is the same as a linear elastic material, while the basic response of a linear dashpot is the same as that of a Newtonian fluid. Combining these two basics in various series and/or parallel arrangements produces the viscoelastic mechanical models, some of which, e.g., Maxwell model, Kelvin model, etc., are too simple to adequately model the actual behaviour of asphalt mixtures, while some other ones, e.g., the Burgers model may properly capture the actual behaviour of the mixture.

The creep compliances were obtained by fitting the test data at different temperatures; the parameters of the four-component Burgers model are listed in Table 1.

### 3.3. Calculation of Relaxation Moduli

Substituting the parameters of the Burgers model into Equation (7), the recovery of the relaxation moduli from the creep compliance at different temperatures is realized. The results are listed in Table 2.

Substituting the data in Table 3 into Equation (4), the relaxation moduli at temperatures 15 °C and 0 °C are obtained:

(11) $$\begin{array}{l} E(t)=2\;032e^{-0.01t}+2\;415.65e^{-1.54t}+3\;742.5e^{-0.011t}+7\;464e^{-0.000\;18t}+5\;645e^{-0.000\;007t}+2\;032e^{-0.000\;000\;5\;t} \end{array}$$

(12) $$\begin{array}{l} E(t)=431e^{-0.001\;5t}+675.65e^{-0.000\;19t}+1\;452.5e^{-0.000\;12t}+431e^{-0.000\;89t}+3\;713e^{-0.000\;000\;3t}+431e^{-0.000\;000\;6\;t} \end{array}$$

## 4. Verification of Calculated Results and Uniaxial Compression Tests

### 4.1. Uniaxial Compression Relaxation Tests

#### 4.1.1. Determination of Constant Levels of Input Strains for Relaxation Tests

The input constant strain is an important parameter for the relaxation test. The input constant strain was obtained for the characterization of the viscoplasticity of the HVAS below the undamaged limit. It has been widely accepted that a sample was not damaged as long as the stress did not reach the vertex of the stress-strain diagram. The property of the mixture changed from linear viscoelasticity to viscoplasticity as the input strain increases during the experiment [33]. Under the condition of small strain, the test specimen was considered not damaged (the strain was in the linear viscoelastic scope), and the constant input strain was assigned conservatively 10% of the strain corresponding to the maximum normal stress. Due to the relaxation nature of the HVAS, the magnitude of the deformation is constant according to the preset program.

#### 4.1.2. Uniaxial Compression Relaxation Tests Procedure

The stress relaxation test is an experiment method to determine the viscoelastic parameters of materials, and it is commonly used to obtain the stress relaxation properties. Since the viscoelastic materials have the memory effect, the stress responses of the materials depend on their loading histories. To this end, the effects of loading histories on the relaxation moduli were investigated. The maximum strain of breaking 20% [34] is recommended to reduce the coefficient of variation in compressive strength tests. Therefore, the designation of the initial strain (0.3 mm) is conservative, that is, 10% of the strain at strength (3 mm).

The direct compression relaxation modulus experiments, at constant input strains of 0.004285, 0.012857, 0.021428, 0.042857, 0.085714, respectively (at input displacements of 0.3 mm, 0.9 mm, 1.5 mm, 3 mm and 6 mm, respectively), and at temperatures of 15 °C, −5 °C and −15 °C, respectively, were carried out on the specimens. The temperature was increased to the predetermined value when the specimens were put inside the environmental chamber. To reach thermal equilibrium in the specimens, it was conditioned for over 4 h. Vaseline was applied on the surfaces of the specimens to reduce the boundary effect and the friction. After the sample was placed between the base and the pressure head of the WDW testing machine (Shanghai Xunrong Testing Equipment Co., Ltd., Shanghai, China), the constant input strain was imposed.

#### 4.1.3. Determination Parameters of GMM Model

The GMM seems to be the best phenomenologic model to represent the HVAS relaxation behaviour [35]. Since the GMM with six arms has the best phenomenologic representation of the viscoelastic behavior of the HVAS, the model was chosen to study the stress relaxation behavior of the HVAS, and the model parameters are listed in Table 3.

#### 4.1.4. Construction of Master Curves for Relaxation Modulus of Asphalt Mixture

The tests were conducted at several temperatures, so a master curve of the relaxation modulus was constructed using the time-temperature superposition principle [36]. The relaxation modulus test protocol is theoretically sound, but practically, the test machine may not be able to control the specimen deformation at a desired constant level. Therefore, the relaxation modulus cannot be simply calculated by dividing the relaxing stress by the strain. Based on the time-temperature equivalence principle, the relaxation modulus curves (RMC) was constructed from short-time relaxation measurements with relevant temperature-shift-factor rates. Williams, Landel and Ferry’s model [37] is used in the analysis:

(13) $$\mathrm{lg}\alpha_{T}=-\frac{C_{1}(T-T_{0})}{C_{2}+(T-T_{0})}=-\frac{C_{1}\Delta T}{C_{2}+\Delta T}$$

where *C*_1_ and *C*_2_ are material parameters and *T*_0_ is the reference temperature, in this study, *T*_0_ = 0 °C.

Shift factors were calculated by the WLF (a formula about time-temperature equivalence principle) to control the relative horizontal displacement at different temperatures as listed in Table 4 [38].

**Figure 8:**
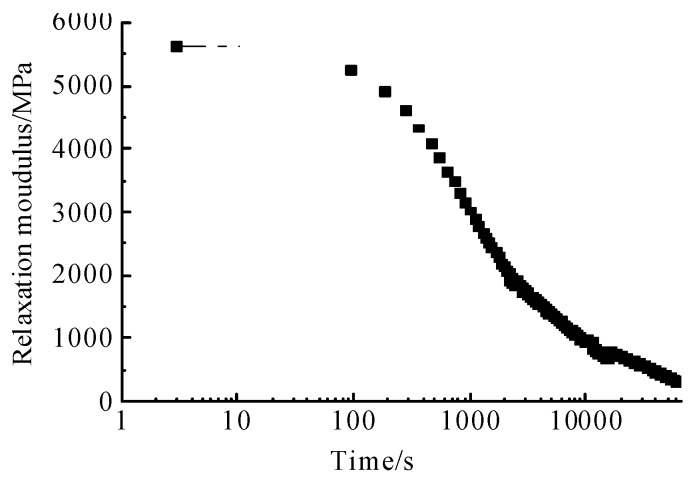
Relaxation master curve at 0 °C.

According to the shift factors given in Table 4, the master curve of stress relaxation moduli was obtained by superposition at 0 °C, as shown in Figure 8.

Figure 8 shows that the stress relaxation rate decreases significantly with the decrease of temperature. In addition, the stress relaxation can be roughly divided into an attenuation stress relaxation stage and a steady stress relaxation stage.

### 4.2. Verification of Calculated Results

**Figure 9:**
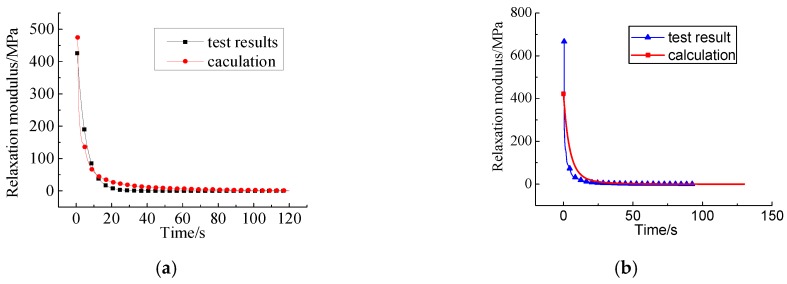

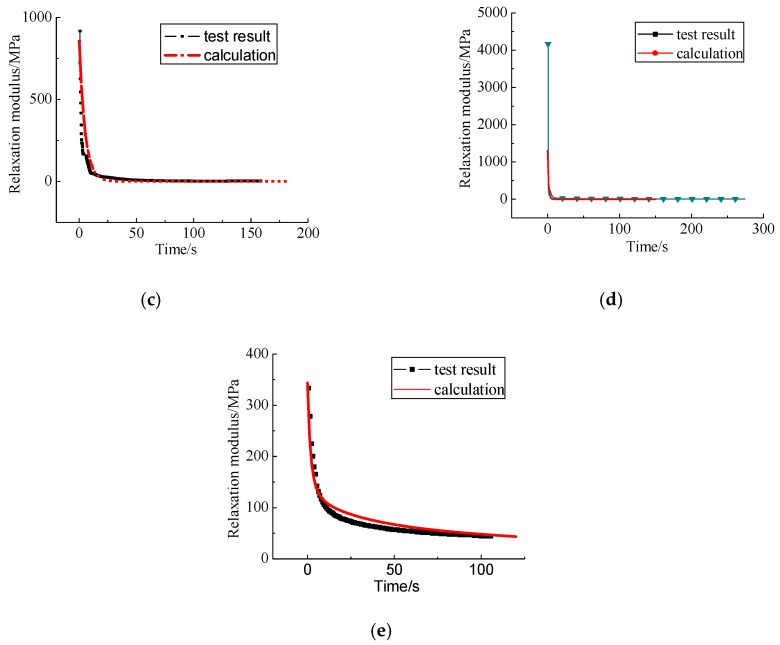
Comparisons of relaxation test results and calculation results at 15 °C. (**a**) 0.3 mm; (**b**) 0.9 mm; (**c**) 1.5 mm; (**d**) 3.0 mm; (**e**) 6.0 mm.

For comparisons, the relaxation moduli by the direct measurements and the calculations from the tests at 15 °C, are shown in Figure 9.

The results show that the relaxation moduli based on the creep tests and the relaxation tests accord with the relaxation properties of the HVAS and have many similar aspects, which indicates that *E*(*t*) can be determined from *J*(*t*) (obtainable from three-point bending creep tests), based on the expansion of the convolution.

The curves of relaxation moduli obtained by the two methods are almost overlapping, but there still exists a narrow margin at the inflection point. This is due to the error between the Prony series and Burgers model in the process of characterizing the viscoelastic properties.

**Figure 10:**
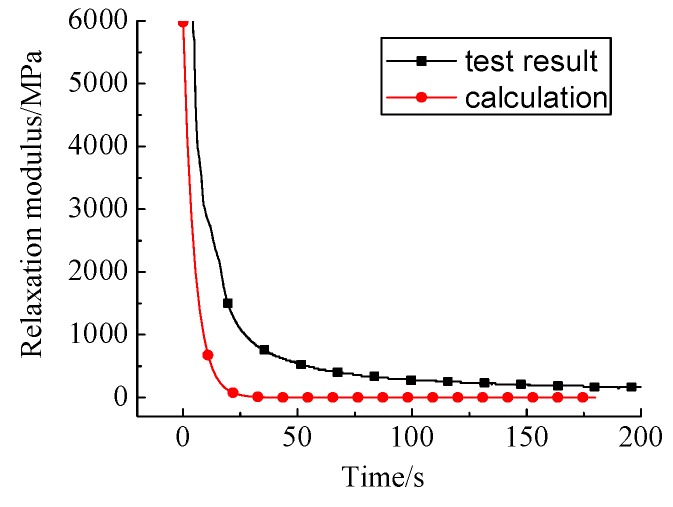
Comparisons of relaxation test results and calculation results at 0 °C.

Similarly, for comparisons, the relaxation moduli by the direct measurements and the calculations from the creep tests at 0 °C are shown in Figure 10.

Compared with the results at 15 °C, the relaxation modulus curves calculated from the two methods display similar trends at 0 °C. Moreover, with the decrease of temperature, the relaxation modulus increases sharply, this proves the validity of the method again. However, there are still some deviations in the relaxation modulus at the inflection point, especially at 0 °C. This is due to the fact that the experimental data of the relaxation modulus were calculated by the WLF formula, and the characteristics of the rheological properties of materials were characterized by the Burgers model.

## 5. Conclusions

The three-point bending creep tests were carried out and the experimental data were fitted by the four-element Burgers model to determine the creep compliance. The *R*-squared values of Burgers are greater than 0.995, which indicates that the model accurately characterizes the viscoelasticity used for the subsequent analysis.

A New Method for the Solving Convolution formula was proposed. To be more specific, the advantages of the Burgers model and GMM are integrated in their respective fields to better characterize the properties of the HVAS.

The creep test is easy to be carried out, which provides creep compliance as the basis for solution. Additionally, the relaxation modulus can be obtained by the transformation relationship between the creep compliance and relaxation modulus. This method avoids the error of the direct relaxation test and reduces the requirement for equipment and operation level.

The method for the recovery of the relaxation modulus from the creep compliance is proposed for solving the relaxation moduli of the HVAS based on the convolution between the creep compliance and the relaxation modulus. In this method, a transcendental equation was obtained by the convolution of the creep compliance and the relaxation modulus, and the polynomial functions were obtained by the expanding of Taylor’s formula, which was solved to obtain the relaxation modulus by Mathmatica using Newton’s method. The method was verified by the relaxation tests.

The results show that the method provides a good agreement between the experiment data and the experimental curves. Therefore, the method can better reflect the relaxation characteristics of the HVAS, and can be used for further study on the relaxation characteristics of the material.

## Figures and Tables

**Table 1:** Burgers model parameters at different temperatures.

Model Parameters	0 °C	10 °C	15 °C
$E_{1}^{\prime }$	3771.931	1893.139	1105.924
$E_{2}^{\prime }$	5996.615	1272.372	705.477
$\eta_{1}^{\prime }$	2.4 × 10^7^	4.2 × 10^6^	2.0 × 10^6^
$\eta_{2}^{\prime }$	5 × 10^6^	1.2 × 10^6^	4.6 × 10^5^
R^2^	0.998	0.999	0.999

**Table 2:** GMM parameters from calculations at 15 °C and 0 °C.

Model Parameters	15 °C	0 °C
*E*_1_/MPa	2032	431
*E*_2_/MPa	2415.65	675.65
*E*_3_/MPa	3742.5	1452.5
*E*_4_/MPa	7464	431
*E*_5_/MPa	5645	3713
*E*_6_/MPa	2032	431
*τ*_1_/s	0.01	0.0015
*τ*_2_/s	1.54	0.00019
*τ*_3_/s	0.011	0.00012
*τ*_4_/s	0.00018	0.00089
*τ*_5_/s	0.0000007	0.0000003
*τ*_6_/s	0.0000005	0.0000006

**Table 3:** GMM parameters at 15 °C.

Model Parameters	0.3 mm	0.9 mm	1.5 mm	3.0 mm	6.0 mm
*E*_1_/MPa	1	1.3	1200	12.21	65.442
*E*_2_/MPa	1.6	1.33	25.56	1	114.98
*E*_3_/MPa	159	106.4	182.06	95.30	54.45
*E*_4_/MPa	341	181.3	94.33	567.09	31.396
*E*_5_/MPa	341.6	181.3	89.3	567309	31.396
*E*_6_/MPa	1	1.3	145.1	1	114.93
*τ*_1_/s	1	1	1	1	1
*τ*_2_/s	1	1	1	18.84	3.16
*τ*_3_/s	12.248	7.63	11.43	18.84	19.5
*τ*_4_/s	1	1	1	1	286.09
*τ*_5_/s	1	1	1	1	296.14
*τ*_6_/s	1	1	1	18.84	3.16

**Table 4:** Temperature shift factors.

Temperature/°C	Temperature Fluctuation ΔT/°C	Shift Factors lg*α_T_*
15	15	1.6636
−5	5	−0.5959
−15	15	−1.8569
